# Re: External validation of HAS model in predicting mortality after emergency laparotomy

**DOI:** 10.1308/rcsann.2025.0127

**Published:** 2026-01-20

**Authors:** S Vijayaraghavalu

**Affiliations:** George Eliot Hospital, UK

Dear Editor,

I read with interest the recent external validation of the HAS model for predicting 30-day mortality following emergency laparotomy.^1^ The reported AUC of 0.90 supports earlier findings that incorporating sarcopenia and physiological reserve can strengthen perioperative risk prediction in acutely unwell surgical patients.^2^ This reflects increasing recognition that frailty and muscle mass strongly influence outcomes in emergency general surgery.

However, several practical issues may limit routine application. The requirement for computed tomography-derived psoas muscle index restricts use in time-critical cases. Many patients with peritonitis, haemodynamic instability or suspected ischaemia proceed directly to theatre without cross-sectional imaging, preventing calculation of the HAS score (Hajibandeh index, American Society of Anesthesiologists status and sarcopenia), whereas the National Emergency Laparotomy Audit (NELA) risk model remains applicable despite accepted limitations.^3^ The modest sample size and exclusion of patients with missing data may also introduce selection bias and restrict generalisability across varied NHS settings.

Although HAS outperformed the parsimonious NELA score in age-stratified analyses, the difference in the full cohort was not statistically significant. This suggests complementary strengths: NELA incorporates operative severity, while HAS provides more detailed physiological profiling. A combined or sequential approach may ultimately offer the most effective predictive strategy.^4^

The HAS model represents a valuable contribution to emergency laparotomy risk assessment, but confirmation through larger, multicentre prospective studies is required to establish clinical usefulness and feasibility in real-world workflows.

Yours sincerely,

Mr Shashikanth Vijayaraghavalu

## Competing interests

The author/s declare no competing interests.

## Funding

The author/s received no financial support for the research, authorship and/or publication of this article.

## Author contributions

**S Vijayaraghavalu:** Visualisation, Writing – review & editing.

## Artificial Intelligence

The author declares that no AI was used to conduct the study or prepare the manuscript.
